# Hunting Levels of Turtle Dove (*Streptopelia turtur*) at Sites Where Food Is Provided: Implications for Sustainable Harvesting

**DOI:** 10.3390/ani12162106

**Published:** 2022-08-17

**Authors:** Gregorio Rocha, Emilio Jorge Tizado, Carlos Sánchez-García

**Affiliations:** 1 Department of Agro-Forestry Engineering, Universidad de Extremadura, 10600 Plasencia, Spain; 2 Department of Biodiversity and Environmental Management, Universidad de León, 24401 Ponferrada, Spain; 3 Department of Research, Fundación Artemisan, 13001 Ciudad Real, Spain

**Keywords:** columbids, European turtle dove, food plots, monitoring, productivity, quota, *Streptopelia turtur*, sustainable hunting

## Abstract

**Simple Summary:**

The European turtle dove (*Streptopelia turtur*) is an important gamebird in south-western Europe, and in some areas a significant proportion of hunting grounds conduct targeted management aiming to increase its breeding densities and hunting opportunities, mainly through food provision. Using harvest data from managed grounds, we estimated the productivity (juvenile/adult ratio), the harvesting levels and the local turtle dove abundance before the hunting season, the latter being compared to the number of birds observed by hunters in food plots. Our research found high values of productivity and significant differences between the estimated abundance and the number of birds observed by hunters, which suggests that in a high proportion of grounds, the latter method may have led to bird overestimation and overharvesting. As managed grounds for the turtle dove may increase the productivity and recruitment of the species, it is crucial to ensure sustainable harvesting through (1) bird monitoring based on transects to calculate abundance and (2) promoting regulations to adjust the number of hunting days.

**Abstract:**

In some regions of Spain, hunting grounds conduct management targeting the European turtle dove (*Streptopelia turtur*), a commonly hunted species, and unsustainable harvesting levels at these sites have been identified as one of the factors responsible for the species’ decline across its range. In hunting grounds where food was provided, we estimated the local turtle dove abundance before the hunting season, productivity (juvenile/adult ratio) and harvesting levels using harvest data from managed grounds over 4 years (2009, 2015, 2019 and 2020). Compared to previous research, a higher productivity value was found (median 1.67, range 1.24–4.15) in grounds providing more food for a longer period. We calculated that the harvesting rate should not exceed 37% of the estimated turtle dove population size (35–45%). Significant differences were found between the estimated local turtle dove abundance using a removal sampling protocol and the number of birds observed by hunters before the hunting season, which suggests that in a high proportion of grounds, the latter method may have led to bird overestimation and overharvesting. Our research supports the current European Union’s harvest management plan to promote sustainable hunting in grounds where targeted management is conducted.

## 1. Introduction 

The European turtle dove (*Streptopelia turtur*, hereafter turtle dove) is a migratory species, wintering in Sub-Saharan Africa and breeding in the western Palearctic [1]. Since the 1970s, populations have declined by 78% [2], owing to poor breeding productivity [3,4] caused by agricultural intensification [5], low survival on wintering grounds [6], habitat loss and degradation, illegal killing and unsustainable hunting [7]. The International Single Species Action Plan for the Turtle Dove (hereafter ISSAP [7]) developed a framework to reverse this population trend using an adaptive harvest management modeling framework, suggesting short-term solutions to food availability and promoting an agri-environment package targeting turtle doves.

In the Iberian Peninsula and other locations, food provision is a common management strategy aiming to compensate for the lack of natural food and increase breeding densities, providing a harvestable surplus and shooting opportunities [8,9]. Surveys indicate that food provision has occurred on 60–90% of hunting grounds in the last two decades [10,11,12]. Typical feeding occurs on plots ~0.2–5.0 ha that are cleared of vegetation, possibly fenced to exclude livestock and big game species (e.g., red deer *Cervus elaphus* and wild boar *Sus scrofa*), and are hand spread with seed mixtures of cereals, legumes and sunflower (*Helianthus* spp.). Managers may also provide water in ponds and troughs, patches of game crops, and other types of habitat improvement through forest management and agri-environmental schemes [12,13]. In 2021, and as part of the adaptive harvest management framework, which is conducted by several European countries in response to the need to ensure sustainable levels of legal hunting along the western flyway [14], hunting was temporarily banned in Spain. Before that, hunting was conducted from mid-August to mid-September, and hunters were required to keep a minimum distance from the plots (200 m in the Extremadura region).

The impacts of food provision and other management actions (such as game crops and water provision) have several population implications. Rocha and Quillfeldt [15] showed that these management actions were associated with higher juvenile/adult bird ratios before the opening of the hunting season when compared to grounds without management. However, this was also associated with a significantly higher proportion of juveniles harvested in these areas [10,15], thus damaging recruitment and population recovery efforts [15]. Hence, a better understanding of management and hunting levels in grounds where food is provided may help address one of the objectives of the ISSAP to ensure that “hunting across the range of turtle doves is carried out at locally and internationally sustainable levels”.

Using data collected during the last decade in hunting grounds of southwestern Spain where food is provided, we aimed to (1) describe how feeding is conducted, (2) address the effects of feeding on the juvenile/adult ratio (age ratio) before hunting and (3) determine whether hunting was sustainable. Our findings may be helpful in the adaptive harvest management framework.

## 2. Materials and Methods

### 2.1. Study Species

**Figure 1:**
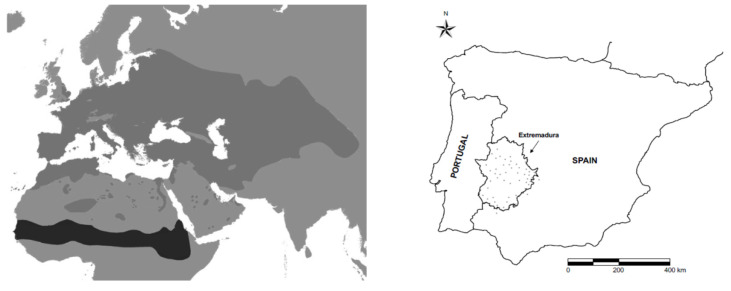
Left: map showing the breeding (dark gray) and wintering (black) distribution of the turtle dove (based on data compiled by Birdlife International, [21]). Right: map of Spain and Portugal showing the region of Extremadura and the locations of the hunting grounds where the study was conducted from 2009 to 2020.

The turtle doves breeding in Iberia are within the western migratory flyway, and prenuptial migration occurs from late March to May, although the majority of birds arrive at their breeding grounds during April and May. Pairs will be formed during migration, but mainly after arriving at breeding grounds, when males will deliver “calls” to attract females [16]. In Spain, 2–3 clutches per pair (with 2 eggs each), will be laid during the breeding season, from April to August. The majority of birds start the post-breeding migration in August–September to the wintering grounds in the Sahel–Sudan zone [7] (Figure 1). The turtle dove is listed as “vulnerable” in Spain and globally [7] and remains a game species in some European countries, but at the moment, it is not hunted in the countries belonging to the western flyway [17].

### 2.2. Study Area

This study was conducted on the hunting grounds located in the historical strongholds of the turtle dove breeding population [18] of the Spanish region of Extremadura but also in the Castilla-La-Mancha and Andalucía regions (Figure 1). In all the hunting grounds, the predominant habitat was the “dehesa”, a savanna-like landscape dominated by evergreen holm oak (*Quercus ilex*) and cork oak (*Quercus suber*) and mixed with grasslands. In this habitat, the main land uses are extensive livestock and wild ungulates for hunting and forestry [19]. The mean size of the hunting grounds is 590 ha (range 300–1200), and the mean altitude is 420 m. The climate is Mediterranean, with hot dry summers, mild winters and an annual mean rainfall of 400 mm [20].

We maintained a minimum distance of 20 km between the selected hunting grounds (i.e., study sites), where food provision had been conducted during spring/summer for at least 10 years before the study began. We aimed to study independent and representative sites of southwestern Spain, ensuring that the type of food provision conducted was the same across sites.

### 2.3. Management and Hunting Practices

We studied 52 different hunting grounds during the whole study period: 49 from Extremadura, 2 from Andalucía and 1 from Castilla-La Mancha (Figure 1). In total, 30 hunting grounds were studied for 1 year, 20 for 2 years and 2 for 3 years (Table 1). There were 25 hunting grounds managed by a local hunting society or syndicate (“social”) and 27 managed by the landowner through a manager or gamekeeper (“private”). Among the latter, 7 were “commercial”; hence, turtle dove hunting days were sold in these grounds. In all hunting grounds, turtle doves were hunted using fixed posts (1 hunter/post), none conducted walk-up hunting and hunting was not allowed within 200 m of food plots, water troughs and ponds. In the grounds studied, other species were hunted: mainly wood pigeon (*Columba palumbus*), rock pigeon (*Columbia livia*) and collared dove (*Streptopelia decaocto*). The latter were legally harvested in some hunting grounds of Extremadura with special permission from the Regional Administration.

Turtle dove hunting was legally practiced in all hunting grounds, and hunting periods varied by year and region. In 2009, in Extremadura and Andalucía, hunting began after 20 August and lasted until the 2nd–3rd week of September, while in Castilla-La Mancha, hunting was allowed from 15 August to 15 September. In 2015 and 2019, in all regions, hunting started on 17 August and lasted until the 2nd–3rd week of September. In 2020, in all studied grounds, hunting was conducted from 29 August to 6 September. The hunting quota (daily bag limit per hunter) ranged from 5 to 15 birds depending on the region and year. Owing to the recent reduction in the hunting periods enforced by administrations, the total number of days in which turtle dove hunting could be conducted was reduced from 10–15 days per season in 2009 to 2–5 days in 2020.

### 2.4. Data Collection

The turtle dove is a species with a large breeding distribution in southern Spain [22]. We considered that the study was conducted on a turtle dove metapopulation, i.e., a group of local populations or subpopulations within the studied areas that may interact [23]. The unit of analysis of this study was the plots where food was provided (1 ha in size, named food plots), not the hunting grounds where these plots were located. In all hunting grounds studied, there was only one plot.

To address the effect of food provision on turtle doves, we recorded the total amount of food provided and the duration of food provision, the latter defined as the period from the first time the food was added to the plot until the start of the hunting season. These data were obtained from meetings and phone interviews with the individuals in charge of game management (hunters and game managers).

To estimate the local abundance of turtle doves before the beginning of the hunting season, we gathered data on turtle dove harvest at the surroundings of the plot where counts were conducted. The data recorded were the number of hunting days, hunting posts (equivalent to hunters) and the total number of turtle doves harvested each day, distinguishing between juveniles (J) and adults (A).

To decide whether harvest could be conducted, local hunters and managers estimated the abundance of turtle doves by counts lasting approximately 1.5 h from the arrival of the first turtle dove (approximately 15–20 min after dawn). The data selected were the maximum number of birds that were simultaneously feeding at the plot during the observation time and the maximum number of birds across the counts when more than one count was conducted. One person carried out the observation with binoculars in the proximity of the plot before dawn [15]. In all plots, at least one count was conducted in the first two weeks of August, from 1 to 7 days before the opening of the hunting season. We were aware that formal transects and call counts during the first hours of the morning in May–June provide a more accurate population estimate [24,25]; hence, counts conducted by hunters/managers could be biased due to variable sampling effort (time and observer number) and lack of consideration of factors influencing the counts. The turtle dove abundance observed per plot by hunters and game managers, close to the beginning of the hunting season, was then compared to the estimated local abundance.

### 2.5. Data Analysis

To estimate the local abundance, we analyzed the game bag harvested in each hunting ground. As 22 hunting grounds were surveyed in more than one year, each hunting ground/year was considered an independent sample (n = 76, Table 1). These data can be regarded as part of a removal sampling protocol, which involves catching and removing animals from a population in several successive time periods (days in this study). The estimated local abundance was calculated using the number of hunted animals and the rate of decline in the consecutive counts. For this analysis, we used those hunting grounds with at least two removal counts (n = 61, range: 2–5).

We were aware of the possibility of a small proportion of turtle doves still breeding in August [11,26], and we could not discount that birds may start their migration to the wintering grounds in August and September, but for the analysis, we assumed that the metapopulation of turtle dove was “closed”, except for those birds removed during the hunting periods. Previous studies have revealed that the age ratio (J/A) can be calculated from hunted birds, although birds hunted from the first day of hunting must be adjusted by a factor of 0.87 to obtain values similar to field age ratios [15].

The hunting effort (fixed post per hunting day) varied among hunting grounds, from 1 to 17 hunting posts (median: 10). To incorporate this hunting effort, we consider a “catch-effort” that assumes a Poisson process allowing regional heterogeneity for removal probability [27]. The “catch-effort” specification considers for each sampling unit (*i*) that the probability of an animal being removed (*p_i_*) is related to the probability that an animal is removed with one unit of effort (*ϕ_i_*) and the effort units applied (*E_i_*, number of hunting posts/hunters):

$$p_{i}=1-{\left(1-\phi_{i}\right)}^{E_{i}}$$

We assumed no differences (*ϕ_i_* = *ϕ*) in the effectiveness of hunters between the hunting grounds, i.e., no per-unit heterogeneity. A Poisson distribution was applied to model local abundance with a different mean of turtle dove per hunting ground (*λ_i_*). To model temporal heterogeneity over years when there is a lack of prior knowledge and relevant covariates, a different mean (metapopulation) abundance per year with a stochastic source variation N (*μ_year_*, *σ*^2^_*year*_) was computed [28]. Then, the final analyzed model was:

$$p_{i}=1-{\left(1-\phi \right)}^{E_{i}}$$

$$X_{i,0}=0,X_{i,t}∼Binomial\left(N_{i}-\overset{t-1}{\underset{j=0}{\sum }}X_{i,j},p_{i}\right)$$

$$N_{i}=Poisson\left(\lambda_{i}\right)$$

$$log\left(\lambda_{i}\right)=\theta_{i}$$

$$\theta_{i}∼Normal\left(\mu_{year\left(i\right)},\sigma_{year\left(i\right)}^{2}\right)$$

Our main goal was to estimate local abundance before removal sampling (*N_i_*), the mean annual metapopulation size (*μ_year_*) and the removal per-unit effort (*ϕ*).

The Bayesian analysis applied to estimate the local abundance was performed using JAGS 4.3.0 [29]. The first 50,000 iterations were treated as a burn-in period, and the following 500,000 iterations with a thinning interval of 20 were saved. The prior distribution used to estimate the per-unit effort was a uniform distribution. The convergence was tested using three chains generated with different initial values [30]. The highest posterior density interval (HPDI) was estimated using an 89% interval [31], while the classical confidence intervals (CI) were 95%. We also used the interval between the 0.25 and 0.75 quantiles (IQi) as a robust estimation of scale.

The harvesting rate (*h_rate_*), including the crippling loss (number of birds hunted but not retrieved), was calculated assuming the same value of winter (not annual) survival of first-year birds and adults, the productivity (p, J/A ratio just before hunting season) and the adult survival (*s_a_*) as follows:

$$h_{rate}=1-\frac{1}{s_{a}\times \left(1+p\right)}$$

We used a generalized linear model (GLM) to test whether local abundances were affected by the type of hunting ground, the province and the amount and duration of food provision [32]. Statistical analyses were conducted using the core packages of R 4.0.4 software (R. C. Team, Vienna, Austria [33]) and the “betareg” package [34].

## 3. Results

**Figure 2:**
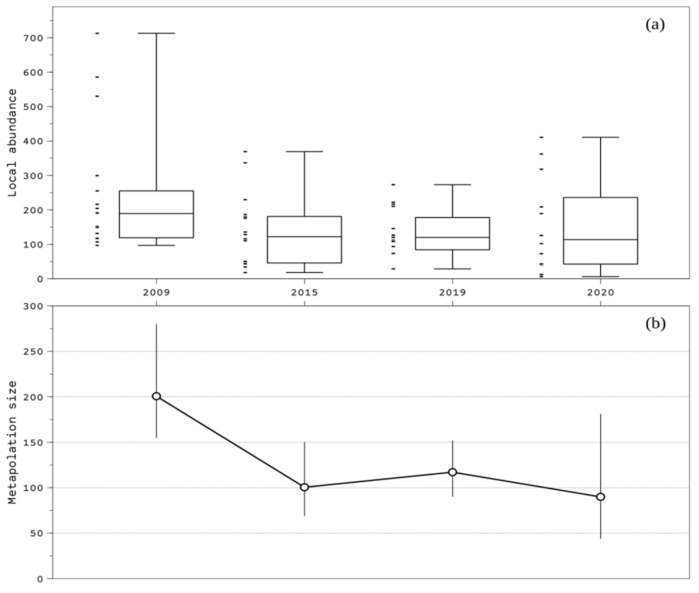
(**a**) Boxplot displaying the interval between the maximum and the minimum value of the estimated local turtle dove abundance in the hunting plots studied in southern Spain (2009–2020) (small bars left to the boxplot show the distribution of sampled data) and (**b**) trend of the mean metapopulation size with standard error bars for the study period (2009–2020).

There was a high variability in the estimated local abundance among hunting grounds and years (ranging from 6.2 to 712.8 individuals, Figure 2a), and we observed a population decline when comparing the results of the metapopulation size from 2009 (median: 200.7 individuals per plot) and 2015, 2019 and 2020 (median: 89.9–117.1 individuals per plot) (Figure 2b). A GLM analysis showed that the estimated local abundance was weakly affected by the type (social/private) of hunting grounds (t = 2.00, *p* = 0.051) and the duration (t = 1.78, *p* = 0.081) of food provision, while it was not affected by the province (e.g., Cáceres vs. Badajoz, t = 0.29, *p* = 0.772) and the amount of food provided (t = −0.40, *p* = 0.691); hence, these were not covariates explaining the estimated local abundance differences among hunting grounds. However, there was strong evidence that commercial hunting grounds were positively associated with higher estimated local abundance compared to noncommercial ones (t = 2.84, *p* = 0.006).

**Figure 3:**
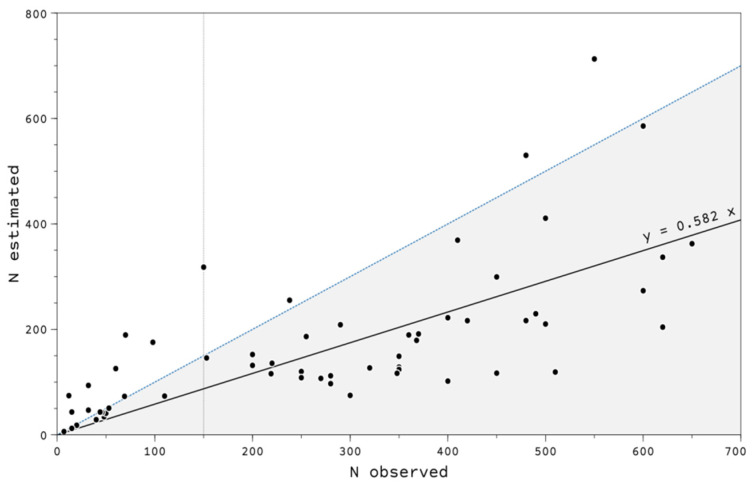
Relationship between the estimated local abundance using the removal sampling protocol (y) and the local abundance observed by hunters/managers (x), in hunting grounds of southern Spain from 2009 to 2020. The solid line shows the non-intercept regression line. The blue dotted line divides the plot between overestimation (gray) and underestimation (blank) areas of the count protocol. The vertical line is the empirical threshold of 150 birds.

Figure 3 compares the estimated local abundance through the removal sampling protocol with the local abundance observed by hunters/managers. Both values showed a significant correlation (R^2^ = 0.79), although the local abundance was overestimated at 71.8% of the plots when applying the observed value instead of the estimated value. However, there was no consistent bias, e.g., using an empirical threshold of 150 turtle doves observed, the local abundance was overestimated in 44.4% of the plots when the number of observed birds was below 150 individuals, whereas this percentage rose to 90.5% when it was higher than 150.

**Figure 4:**
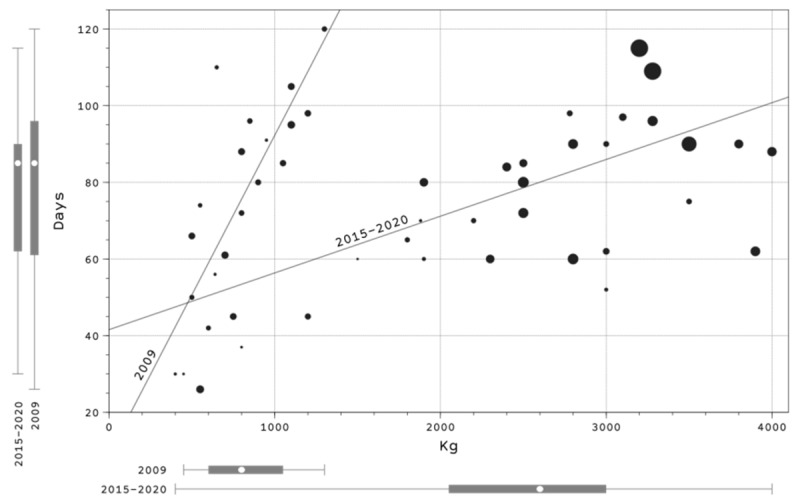
Relationship between the amount of food provided and the duration of food provision in hunting grounds managed for turtle dove from southern Spain, distinguishing two periods: 2009 and 2015–2020. The diameter of the points is proportional to the log-value of the productivity. The solid lines are the regression lines. The small bars in the margins of the plot area show the minimum, Q1, median, Q3 and maximum of each distribution.

In 2009, the feeding conducted at the plots lasted from 26 to 120 days (median: 85 days), with a maximum amount of 1300 kg provided per plot (median: 800 kg); hence, the mean amount of food provided was 11.38 kg/day. For the period 2015–2020, higher amounts were recorded (median: 2600 kg) during similar periods of 30–115 days (median: 85 days, median: 28.89 kg/day, Figure 4).

We analyzed the effect of food provision in relation to the age ratio immediately before the beginning of the hunting season. The results showed a mean productivity of 1.85 before the hunting season (median: 1.67, range: 1.24–4.15), with half of the plots showing a value ranging from 1.52–2.03 (IQi). The GLM showed no evidence of effects of either variable on productivity (duration of food provision: t = −1.31, *p* = 0.198, amount of food provided: t = −1.78, *p* = 0.082). However, very strong evidence of a positive interaction was found (t = 2.87, *p* = 0.006) between both variables; hence, more food provided during a longer period increased productivity.

The number of hunters per day ranged from 3 to 17, although 50% of the hunts had between 8 and 12 hunters (median: 10). The number of hunting days per season ranged from 1 to 5 days, although in 75% of hunting grounds hunting was conducted from 1 to 3 days (median: 2 days). The median number of turtle doves harvested per hunting ground was 101.5, and 50% of the grounds harvested between 47 and 175 individuals, while only 25% harvested more than 175 birds per season. The mean number of turtle doves harvested per hunter and day was 5.28 (median: 4.44, IQi = 2.75–7.06). Seventy-five percent of the hunting grounds harvested less than half of the maximum allowable (15 turtle doves), and only two hunting grounds (3% of the total studied) harvested the maximum.

**Figure 5:**
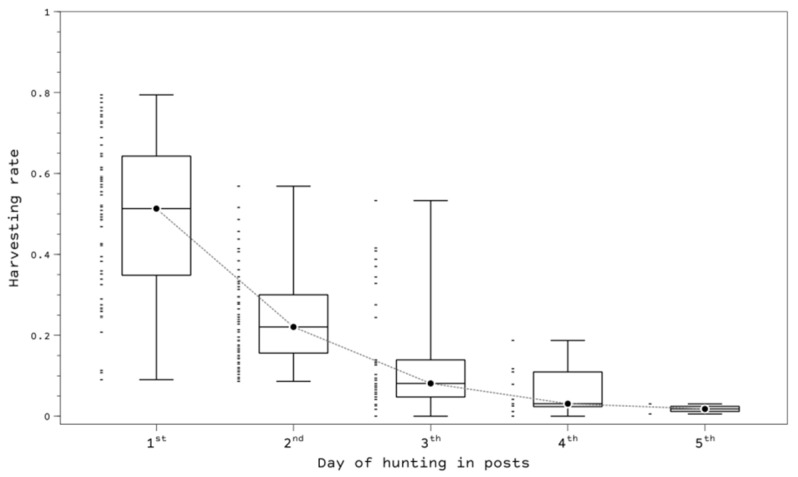
Harvesting rate of the turtle dove in relation to the day of hunting. Black dots are median values, and small bars to the left of the boxplot show the distribution of the sampled data. To highlight the trend, a dotted line connecting the medians is shown.

The analysis of the hunting bag in relation to the estimated local abundance showed a high harvesting rate, as half of the hunting grounds (IQi) harvested between 78.4% and 93.2% (median: 84.0%), and 20% of the grounds hunted less than 75% of the estimated local abundance. The harvesting rate was influenced by hunting days in relation to the remaining population size (Figure 5), e.g., during the first hunting day, 51.3% of the estimated local abundance was hunted (IQi: 34.8–64.3%). At commercial hunting grounds, the harvesting rate was 9% higher (median: 90.8%) than that at noncommercial grounds (median: 83.3%).

**Figure 6:**
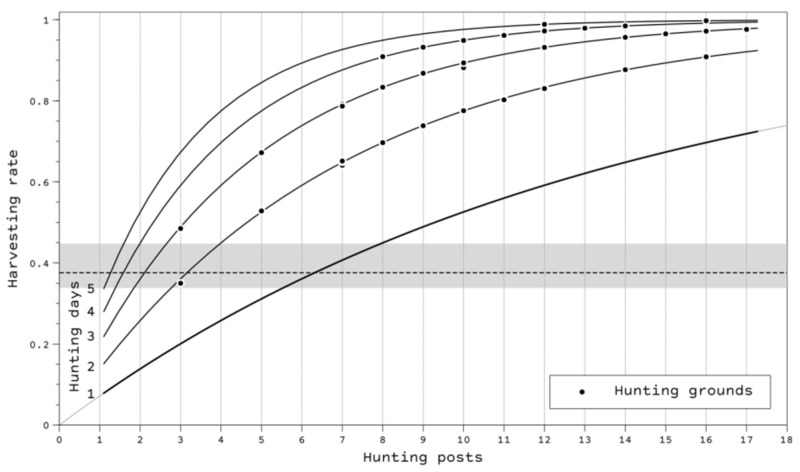
Relationship between the harvesting ratio and the number of hunting days and posts (equivalent to the number of hunters) at hunting grounds in southern Spain. The dashed line shows the maximum harvest rate to ensure sustainable hunting per season (the gray area is the IQ interval), e.g., 1 day of hunting with 6–7 posts (hunters) and 2 days with 2–3 posts.

The beta regression analysis of the harvest rate showed significant positive effects from the number of hunting posts and the number of hunting days (with a value of 0.955 to the pseudo-R^2^). However, the effect of hunting days was 1.54 higher than the number of hunters; each additional hunting day increased the harvest by 20.2%, whereas the effect of adding an additional hunter increased the harvest by 14.1%. Figure 6 shows the relationship between these variables using a median removal per-unit effort (*ϕ*) of 0.072 (HPDI: 0.070–0.074). The proposed harvest rate (e_rate_) calculated to ensure long-term sustainable hunting was 37.21% (IQi: 33.78–44.92%), using an annual adult survival of 0.597 (Bacon et al., 2020) and age ratio at the studied plots of 1.67 (IQi: 1.52–2.03).

## 4. Discussion

Our results showed that the productivity of turtle doves on hunting grounds where food was provided was 1.67 (IQi 1.52–2.03). Assuming the same winter survival value for juveniles and adults, this result is 3.04 times higher than the 0.54 calculated productivity value for the general population [35], using a fecundity of 1.035 (age ratio) and annual survival values of 0.311 for juveniles and 0.597 for adults. Our results suggest that food provision increases productivity and contributes to higher recruitment before the hunting season. A study conducted in Britain [36] found that turtle dove nesting success and chick biometrics were unrelated to the local availability of seed-rich habitats provided through plots. However, nestling weight was higher when birds were closer to “anthropogenic food”. In the Extremadura region [15], it was shown that food provision targeting turtle doves induced changes in abundance and breeding success, reducing the detrimental effects of limited food resources, such as reduced breeding performance and earlier termination of the breeding season [37].

The duration of food provision was similar during the study period (median: 85 days), but the amount provided per plot increased from 800 kg in 2009 to 2600 kg in recent years. This may be attributed to the hunting interest in turtle doves and other columbids in this part of Spain, the lack of natural food experienced in the last decade, caused by changes in agriculture [38] or lack of management of forested areas which may result in low availability of natural habitats in which turtle doves find seeds [39]. In some cases, food provision is the only way to provide food due to the difficulties in creating seed-rich habitats (such as uncultivated plots and game crops) [12]. Although the food provided at the plots targeted columbids, hunters and game managers are aware of other species taking the grain (such as red-legged partridge *Alectoris rufa*, wild boar and red deer).

Although we chose independent and representative sites (ensuring that management traits were the same across them), the estimated local abundance at the studied plots varied. This is possibly explained by differences in carrying capacity and habitat favorability [22], together with different levels of intensity of game management [12], such as the duration and the amount of food provided. It is then possible that the higher local abundance estimated for the commercial hunting grounds could be related to higher levels of management and more favorable habitat for turtle doves, and further studies should address this question.

The trend of the metapopulation size observed for the study period (2009–2020) showed a reduction from 2009 to 2015 and a certain degree of stabilization from 2015 to 2020. Although our study was carried out in different hunting grounds from a specific geographical area, and the methodology focused on addressing the harvesting levels of turtle doves at hunting grounds providing food, our results are similar to the population trend observed for turtle doves in Spain in recent years, showing a decline until 2012–2014 but changing toward relative stabilization in later years [22].

We found significant differences between the number of turtle doves observed at the plots before the hunting season and the estimated local abundance using the removal sampling protocol. When the number of turtle doves counted by hunters/game managers was below 150, there was an underestimation of the population in most cases. In contrast, the opposite occurred when it was above 150 birds, obtaining values 172.4% higher than those calculated through the sampling removal protocol. This could be explained by the fact that bird counts at the plots were observational data without a replica, with no constant bias, which hampers the correction of this methodology. It is likely that in those plots with higher numbers of turtle doves counted, bird counts were influenced by the influx of birds from the areas surrounding the plots but also coming from other more distant areas. It is known that turtle doves may conduct significant movements in search of food [36].

The hunting levels varied significantly on the hunting grounds. On average, hunting was conducted for 2 days, and 10 hunters participated per day, although in some plots, no hunting was conducted, and in other plots, hunting was conducted for 5 days, with up to 17 hunters per day. As recorded in other countries where turtle doves are hunted [40], the highest harvesting rate was reached in the first week of the hunting season, which is not surprising. The overall harvest rate was high, especially on commercial hunting grounds, and although we do not know the effects of the harvest rate on a given turtle dove population (because our unit of analysis was the local abundance at the plot), it is likely that in some hunting grounds, overharvest scenarios occurred, which could be detrimental for turtle dove conservation [15]. As the counts conducted by hunters/game managers overestimated local abundances and the quotas to ensure sustainable hunting in each ground may not have been effective, it is necessary to promote monitoring within the hunting community through citizen science projects at a broader scale owing to the migratory behavior of turtle doves (www.observatoriocinegetico.org (accessed on 23 April 2022) and efficiently implement hunting regulations. Further research is needed to address hunting patterns in Extremadura, the region where most of the grounds were located. A proportion of the official bag data for the 2020 hunting season (n = 1033 hunting grounds, one-third of the total number) shows that turtle dove hunting was declared in 36% of hunting grounds, and no hunting was conducted in 55% of the surveyed territory (from a total hunting area of 3.47 million ha).

Our results confirm that when aiming to adjust turtle dove hunting levels, reducing the number of hunting days is more effective than reducing the number of hunters. Previous studies of migratory species have shown that shortening the hunting season results in lower hunting levels; this is not the same for sedentary species [41,42]. In Spain, a recent study suggested that hunting regulatory mechanisms conducted from 2007 to 2017 to reduce harvesting levels of turtle doves were ineffective in substantially decreasing the game bag [43]. A study conducted in Castilla-La Mancha using data from hunting management plans suggested reducing hunting intensity by reducing hunters per unit area or the availability of fixed-positions hunting days [44].

## 5. Conclusions

Hunting grounds providing food for turtle doves may increase the productivity of the species, although hunting levels should be revised to avoid overharvesting scenarios. Furthermore, to ensure accurate population estimates and productivity, we recommend turtle dove monitoring based on scientifically adjusted transects and listening for singing males during the breeding season at a broad spatial scale. Data gathered in plots where food is provided should be treated with caution, especially where the number of turtle doves counted is high (e.g., higher than 150 individuals), leading to an overestimation of the population which ultimately will result in overharvesting. We propose that once the population size for an area with this type of management has been estimated, the harvesting rate should not exceed 37% of the stock (range 35–45%). When aiming to achieve this harvesting rate in grounds where food is provided, policy makers should promote regulations to adjust the number of days of both hunting and hunters per day, 1 day of hunting with a maximum of six to seven hunters, or 2 days with a maximum of two to three hunters per day.

## Figures and Tables

**Table 1:** Number of hunting grounds studied per region, year and type. The commercial hunting grounds are shown in brackets.

	2009		2015		2019		2020		Total	
Region	Social	Private	Social	Private	Social	Private	Social	Private	Social	Private
Extremadura	9	11 (5)	9	8 (2)	6	14 (2)	6	10 (2)	30	43 (11)
Castilla-La Mancha	0	0	0	1	0	0	0	0	0	1
Andalucía	0	0	1	1	0	0	0	0	1	1
Subtotal	9	11	10	10	6	14	6	10	0	0
Total	20		20		20		16		31	45

## Data Availability

The data used in this study is available upon reasonable request.
